# Plant-Mediated Rhizosphere Oxygenation in the Native Invasive Salt Marsh Grass *Elymus athericus*

**DOI:** 10.3389/fpls.2021.669751

**Published:** 2021-06-10

**Authors:** Ketil Koop-Jakobsen, Robert J. Meier, Peter Mueller

**Affiliations:** ^1^Wadden Sea Station, Alfred Wegener Institute, Helmholtz Centre for Polar and Marine Research (AWI), List/Sylt, Germany; ^2^PreSens Precision Sensing GmbH, Regensburg, Germany; ^3^Institute of Soil Science, Center for Earth System Research and Sustainability, Universität Hamburg, Hamburg, Germany

**Keywords:** tidal wetland, plant-soil interaction, sediment oxygenation, ROL, wetland plants, aerenchyma, planar optode, imaging

## Abstract

In the last decades, the spread of *Elymus athericus* has caused significant changes to the plant community composition and ecosystem services of European marshes. The distribution of *E. athericus* was typically limited by soil conditions characteristic for high marshes, such as low flooding frequency and high soil aeration. However, recently the spread of *E. athericus* has begun to also include low-marsh environments. A high-marsh ecotype and a low-marsh ecotype of *E. athericus* have been described, where the latter possess habitat-specific phenotypic traits facilitating a better adaption for inhabiting low-marsh areas. In this study, planar optodes were applied to investigate plant-mediated sediment oxygenation in *E. athericus*, which is a characteristic trait for marsh plants inhabiting frequently flooded environments. Under waterlogged conditions, oxygen (O_2_) was translocated from aboveground sources to the roots, where it leaked out into the surrounding sediment generating oxic root zones below the sediment surface. Oxic root zones were clearly visible in the optode images, and no differences were found in the O_2_-leaking capacity between ecotypes. Concentration profiles measured perpendicular to the roots revealed that the radius of the oxic root zones ranged from 0.5 to 2.6 mm measured from the root surface to the bulk anoxic sediment. The variation of oxic root zones was monitored over three consecutive light–dark cycles (12 h/12 h). The O_2_ concentration of the oxic root zones was markedly reduced in darkness, yet the sediment still remained oxic in the immediate vicinity of the roots. Increased stomatal conductance improving the access to atmospheric O_2_ as well as photosynthetic O_2_ production are likely factors facilitating the improved rhizosphere oxygenation during light exposure of the aboveground biomass. *E. athericus’* capacity to oxygenate its rhizosphere is an inheritable trait that may facilitate its spread into low-marsh areas. Furthermore, this trait makes *E. athericus* a highly competitive species in marshes facing the effects of accelerated sea-level rise, where waterlogged sediment conditions could become increasingly pronounced.

## Introduction

*Elymus athericus* (Link) Kerguélen (henceforth referred to as *Elymus*) is markedly increasing its areal coverage in NW European salt marshes, altering the characteristic plant community composition and becoming a dominant plant species (Leendertse et al., 1997; van Wijnen et al., 1997; Valéry et al., 2017; Hartmann and Stock, 2019). This spread has been described as one of the most significant changes of the NW European salt-marsh landscape in the last decades (Valéry et al., 2004). The spread of *Elymus* may significantly impact the ecosystem services that the salt marshes provide altering the sedimentation and carbon storage capacity (Valéry et al., 2004; Hartmann and Stock, 2019; Nolte et al., 2019) and changing the marshes’ role as a nursery for the coastal fish populations (Laffaille et al., 2005).

*Elymus* is primarily confined to the high marsh, characterized by lower inundation frequency (Bockelmann et al., 2002) and higher soil aeration (Armstrong et al., 1985). The spread of *Elymus* in the high marsh has, in part, occurred in response to the abandonment of grazing practices and increased man-made drainage activities (Rupprecht et al., 2015; Hartmann and Stock, 2019), as well as increased sedimentation enhancing the surface elevation (Nolte et al., 2019). Increased nitrogen loading may also be a driver for the spread of *Elymus* in European marshes (Olff et al., 1997; Van Wijnen and Bakker, 1999; Rozema et al., 2000; Valéry et al., 2017). This is, however, not always the case (Bockelmann and Neuhaus, 1999; Veeneklaas et al., 2013; Nolte et al., 2019).

Strikingly, the spread of *Elymus* is not restricted to the high marsh, and progressive invasion into the low-marsh environments has recently been observed in various studies (Olff et al., 1997; Veeneklaas et al., 2013; Valéry et al., 2017; Nolte et al., 2019; Mueller et al., 2021; Reents et al., 2021). An important factor likely contributing to *Elymus*’ competitiveness across a large range of flooding frequencies is the presence of discrete *Elymus* genotypes from high-marsh and low-marsh environments, respectively. These genotypes are reflected in phenotypic characteristics (Bockelmann et al., 2002, 2003). The low-marsh ecotype is characterized by having habitat-specific phenotypic traits, such as higher aboveground biomass production, longer rhizomes, shoots, and leaves, which facilitates a better adaption for inhabiting areas with higher flooding frequencies (Chen, 2020; Reents et al., 2021). Overall, however, the morphological and physiological mechanisms behind *Elymus*’ high competitiveness in these frequently flooded environments are poorly understood (Mueller et al., 2021).

The low marsh is frequently flooded by seawater at high tide, and the sediment is primarily waterlogged resulting in anoxic conditions and sulfide accumulation. The marsh plants that inhabit the low marsh have adaptive traits, which enable them to cope with these harsh living conditions (Parent et al., 2008; Pan et al., 2020). Well-developed aerenchyma is a key trait facilitating a rapid supply of oxygen (O_2_) from aboveground sources to the belowground roots and rhizomes, where it may leak out and oxygenate the surrounding sediment, generating oxic root zones below the sediment surface (Colmer, 2003; Sorrell and Brix, 2013). Plant-mediated sediment oxygenation is a trait that reduces the phytotoxic impact of sulfide accumulation (Lee et al., 1999; Pezeshki, 2001) and improves nutrient uptake (Bradley and Morris, 1990; Lai et al., 2012). We hypothesize that the *Elymus* low-marsh ecotype, in order to thrive and be competitive in the low marsh, possesses the ability to transport O_2_ from aboveground sources to its roots and further into the sediment. In the high marsh, belowground transport of O_2_ is not a necessity, as the soils are typically well-drained and aerated (Mueller et al., 2020). In many wetland plants, the porosity of the roots is often lower in plants grown under drained/aerated conditions than plants grown under waterlogged and O_2_-deficient conditions (Colmer, 2003). Hence, we hypothesize that the capability of transporting O_2_ during events of flooding is limited in the *Elymus* high-marsh ecotype.

In this study, we investigated the formation of oxic root zones during sediment waterlogging in the low-marsh and high-marsh ecotype of *E. athericus*. Planar optodes were applied to image the O_2_ haloes evolving around individual roots in the rhizosphere. The effect of light availability of the aboveground biomass on belowground sediment oxygenation was investigated, monitoring oxic root-zone formations during consecutive light–dark cycles.

## Materials and Methods

### Experimental Design

Plant-mediated sediment oxygenation was investigated by imaging oxic roots zones in four replicate plant samples of the low-marsh and high-marsh ecotype, respectively. The impact of light on belowground O_2_ dynamics was investigated, monitoring the oxic roots zones during three consecutive light/dark cycles (12 h/12 h). The spatial extension of oxic root zones was measured as O_2_ profiles across selected oxic root zones. Both *Elymus* ecotypes were investigated under waterlogged conditions in order to distinguish roots leaking O_2_ into the anoxic sediment.

### Plant Samples and Cultures

Plants were collected, the Netherlands in April 2015 on the Wadden Sea island of Schiermonnikoog. The sampling was conducted in stands that previously were demonstrated as being genetically distinct populations of *Elymus*, i.e., high-marsh and low-marsh ecotypes (Bockelmann et al., 2003). The two ecotypes were grown under identical conditions in a common-garden setting exposed to natural changes in precipitation and sunlight at the University of Hamburg for 5 years before investigations commenced. Low-marsh and high-marsh ecotypes were still phenotypically distinct after 5 years (Mueller et al., 2021; Reents et al., 2021), which confirms that the morphological differences between the low-marsh and high-marsh ecotypes are indeed caused by genetic differences (Mueller et al., 2021; Reents et al., 2021), as described by Bockelmann et al. (2003).

### Planar Optode Imaging

**Figure 1:**
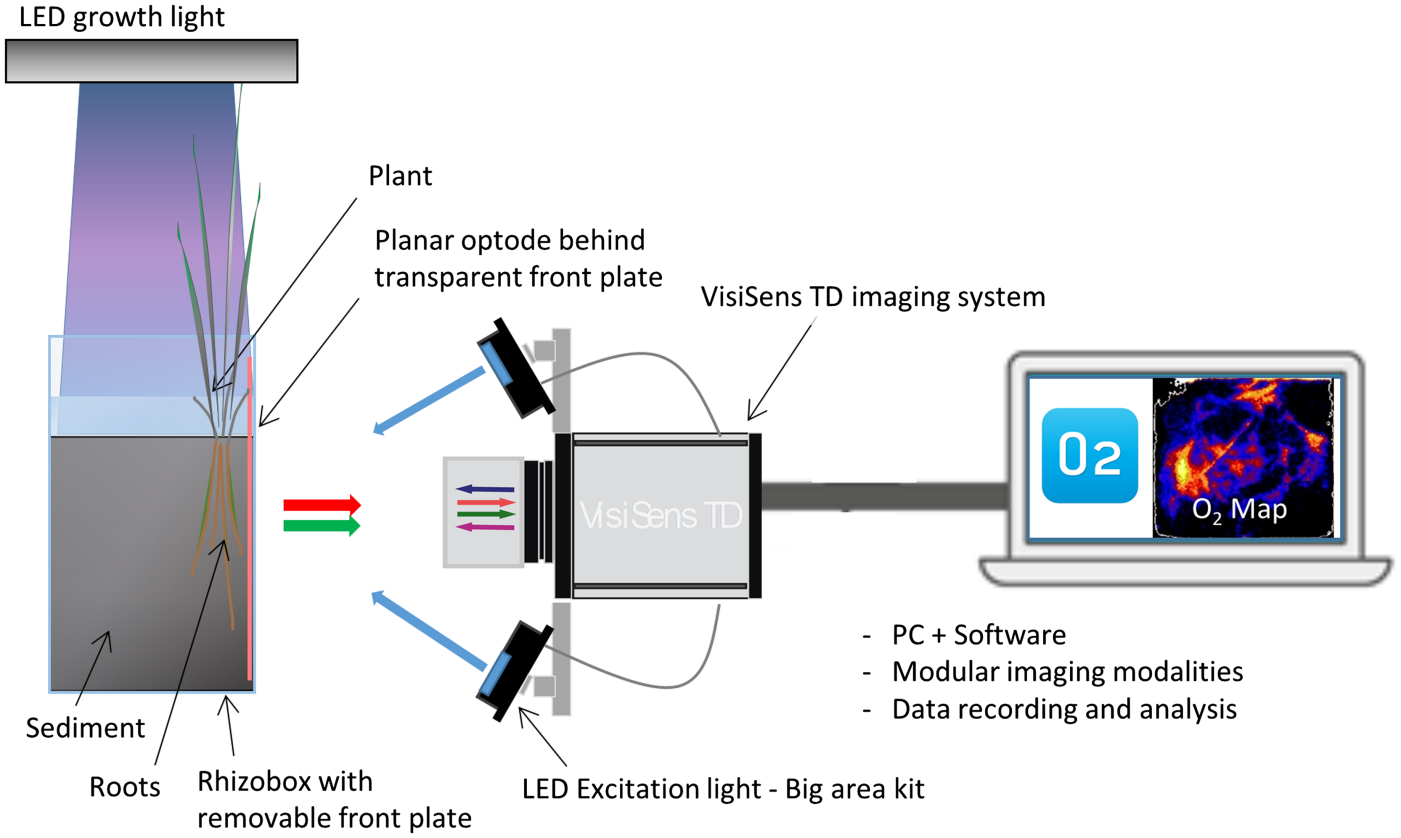
Experimental setup for planar optode investigations of rhizosphere processes with the planar optode system VisiSens TD. A rhizobox with sediment and the sample plant is equipped with a planar luminescent O_2_ sensor foil. The aboveground biomass of the plant is illuminated in a day–night cycle with a LED growth light. The O_2_ dependent signals of the sensor foil are read out with the VisiSens camera detector under excitation with specific blue excitation light. The recorded signals are transformed into 2D O_2_ maps *via* the camera software.

The formation of oxic root zones in the rhizosphere of *Elymus* was investigated using the planar optode system, VisiSens TD from PreSens – Precision Sensing GmbH, which is a ratiometric optode imaging system allowing for 2D imaging of O_2_ (pH and CO_2_) distributions. The O_2_ optode imaging is based on the dynamic quenching of a luminophore in the presence of O_2_. For 2D imaging of plant-mediated sediment oxygenation of the rhizosphere, the plant sample for investigation is placed in a rhizobox with roots facing the transparent front plate of the box. An optode foil, coated with a luminescent O_2_-sensitive dye, is placed on the inside of the rhizobox in direct contact with the roots and sediment. The foil is excited by a LED light source, and the luminescent response is recorded with a camera. Through calibration, each pixel in the image is assigned an O_2_ value, generating a quantitative 2D image of the O_2_ distribution (Figure 1). The limit of detection for the VisiSens TD system with SF-RPFu4 O_2_ sensing foils is 0.03%.

For a detailed description of the theoretical background behind the ratiometric optode imaging principles used in the VisiSens optode system, we refer to Wang et al. (2010), Tschiersch et al. (2011), and Tschiersch et al. (2012).

### Preparation of Plant Samples for Planar Optode Investigations

In preparation for planar optode investigations, single shoots were separated 3 months prior to the optode measurements and cultured in a substrate consisting of 70% sand and 30% potting soil. Salinity was reduced to zero to ensure recovery of shoots after separation. The low-marsh ecotype was kept under permanently waterlogged conditions, and the high-marsh ecotype was kept under moist sediment conditions with regular watering.

For the preparation of a rhizobox with *Elymus* roots placed up against the transparent front plate, the coarse organic matter was washed out of the substrate, and the remaining sandy sediment was slurred and filled in a rhizobox (10 cm × 10 cm × 10 cm) with a detachable front plate. The sediment was allowed to settle for a minimum of 1 day. These procedures were installed to get a uniform sediment with high stability, which allowed for the rhizobox to be turned to a 45° angle and the front plate to be removed, without the sediment collapsing, exposing a well-defined vertical sediment surface. In this way, the roots and rhizomes of the plant sample could manually be distributed over the vertical sediment surface, facing the front plate, allowing for direct contact with the optode foil.

Subsequently, an O_2_-sensitive optode foil (SF-RPSu4; 10 cm × 15 cm; range, 0–100% O_2_ atm. sat., PreSens GmbH) was attached to the inside of the detached front plate of the rhizobox. Both the front plate and optode foil were placed in a container with water. Under water, the optode foil was pressed up against the front plate, and both items were lifted out of the water together. In this way, a uniform and air-bubble-free water film was generated between the foil and the front plate, holding the foil in place.

The front plate with foil was attached back on the rhizobox, and the rhizobox was then slowly moved back to an upright position, while water was simultaneously added along with the back plate. In this way, the sediment along the front plate was slowly saturated with water from the bottom up, minimizing the entrapment of air bubbles along with the optode foil. The water-filled rhizobox had a 3–5 cm water column on top of the sediment with the adult *Elymus* plants being emergent through the water column.

### Experimental Setup

Optode imaging of the rhizosphere O_2_ distribution was conducted simultaneously on two rhizoboxes, using external LED excitation lights (Big Area Imaging kit, PreSens GmbH). During light periods, the aboveground biomass was illuminated with a LED growth light (Roleadro 270 W LED) placed 40 cm above the plants. Prior to the optode measurements, the sample-prepared rhizobox was run through a minimum of two light–dark cycles, reestablishing normal sediment conditions after the sample preparation. The light conditions under the growth light were too bright for optode imaging, which requires conditions without ambient light. In order to enable optode recordings during the light period, the illumination of the aboveground biomass was turned off for a 3-min period every 30 min, while each optode image was acquired. The optode investigations were conducted in freshwater within a temperature range from 18 to 20°C. At the end of each optode experiment, the front plate with optode foil was detached, and the vertical sediment surface was photographed showing the relative position of the roots. In cases where many roots were partially hidden behind the sediment, the sediment surface was gently rinsed with droplets of water from a Pasteur pipette in order to expose the roots and make them visible in the image.

### Image Analysis

The planar optode images were analyzed using the VisiSens ScientifiCal software, which is an integrated part of the optode imaging system. The acquired optode images had a resolution of ~5 pixels per millimeter at the chosen field of view. The internal noise filter was applied to smoothen the optode images with kernel size 2. A two-point calibration was applied, using air-bubbled water for 100% atmospheric equilibrium O_2_ (100% atm. sat.) and anoxic sediment for 0% atm. sat. The calibration converted the sensor readout into quantitative 2D images of the O_2_ distribution. O_2_ concentrations were expressed as the percentage of O_2_ saturation at atmospheric equilibrium (% atm. sat.).

For investigation of the impact of light availability on the temporal distribution of oxic root zones, the average O_2_ concentration was measured continuously in specific areas of each sample, representing an oxic root zone and the bulk anoxic sediment, respectively. The oxic root zone area was manually selected as a root zone with oxygenation below the sediment surface clearly distinguishable from the bulk anoxic sediment, and likewise, the anoxic bulk sediment area was selected as an anoxic zone without roots below the sediment surface clearly distinguishable from the oxic root zones. This way of measuring temporal variation was conducted using the ScientifiCal software, which allows for the average concentration to be followed over time within a manually selected area of the optode foil.

For investigation of the impact of light availability on the spatial distribution of oxic root zones, cross-sectional concentration profiles were measured perpendicular to selected O_2_ leaking roots under light and dark conditions. One profile was measured per sample. Profiles were selected from areas where O_2_ leaking from single roots was clearly distinguishable from the background O_2_ concentration. In cases with more selectable roots, the root with the highest O_2_ concentration was chosen. Larger oxic root zones most often had multiple roots contributing to O_2_ leakage; these areas were avoided. This spatial profiling was conducted using the ScientifiCal software (Live Profile plugin). The width of the roots was measured directly from images of the rhizobox using the open-source software ImageJ Fiji (1.53c).

## Results

### Oxic Root Zones in Low- and High-Marsh Ecotypes

**Figure 2:**
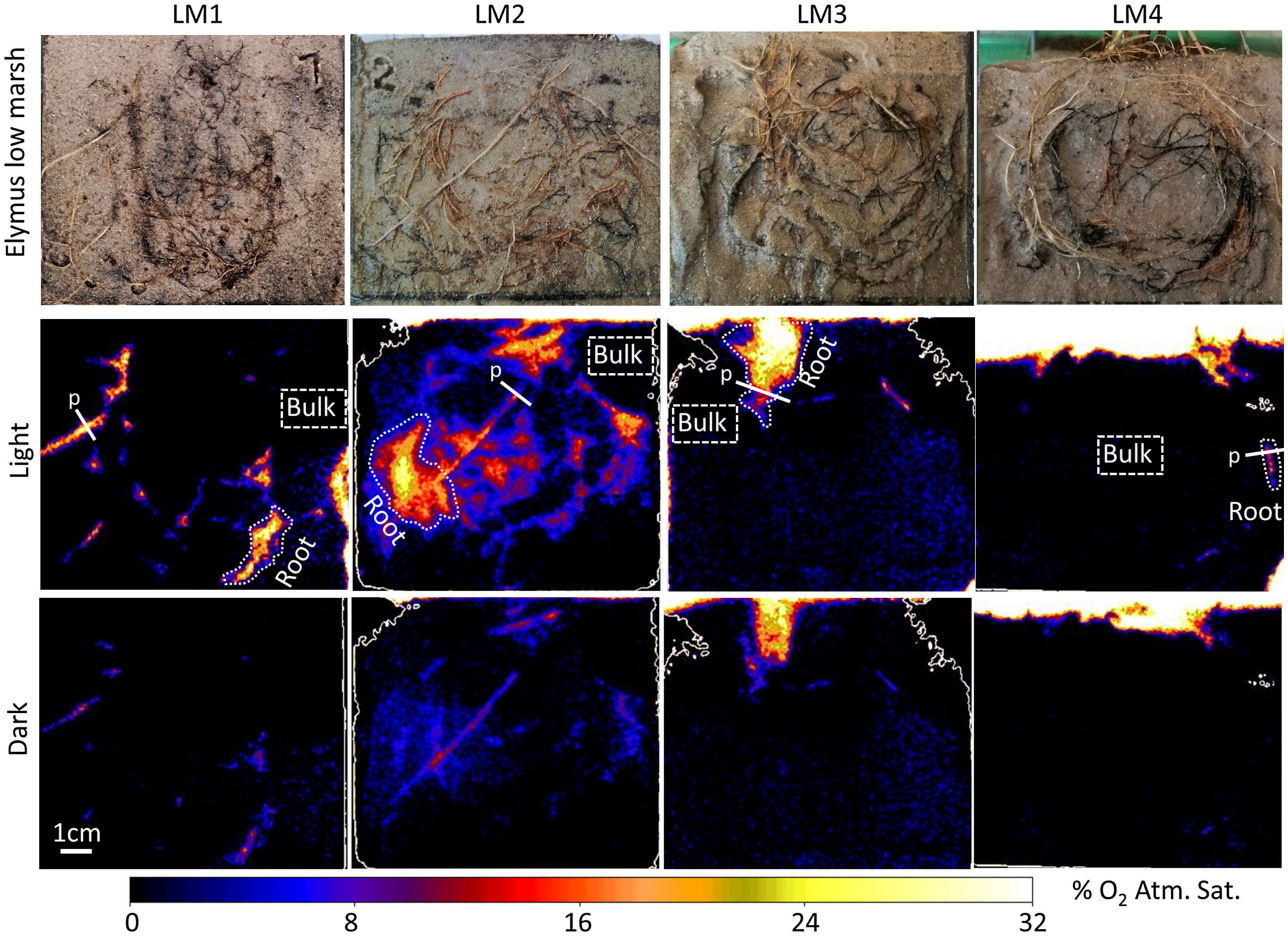
Planar optode images of the O_2_ distribution around selected roots of *Elymus athericus* – low-marsh ecotype from four independent samples (LM1–LM4), with and without illumination of the aboveground biomass. “Root” indicates specific oxic root zones, and “Bulk” indicates specific areas with bulk anoxic sediment selected for quantitative measurements shown in Figure 4. “p” indicates the locations of the concentration profile shown in Figure 5.

**Figure 3:**
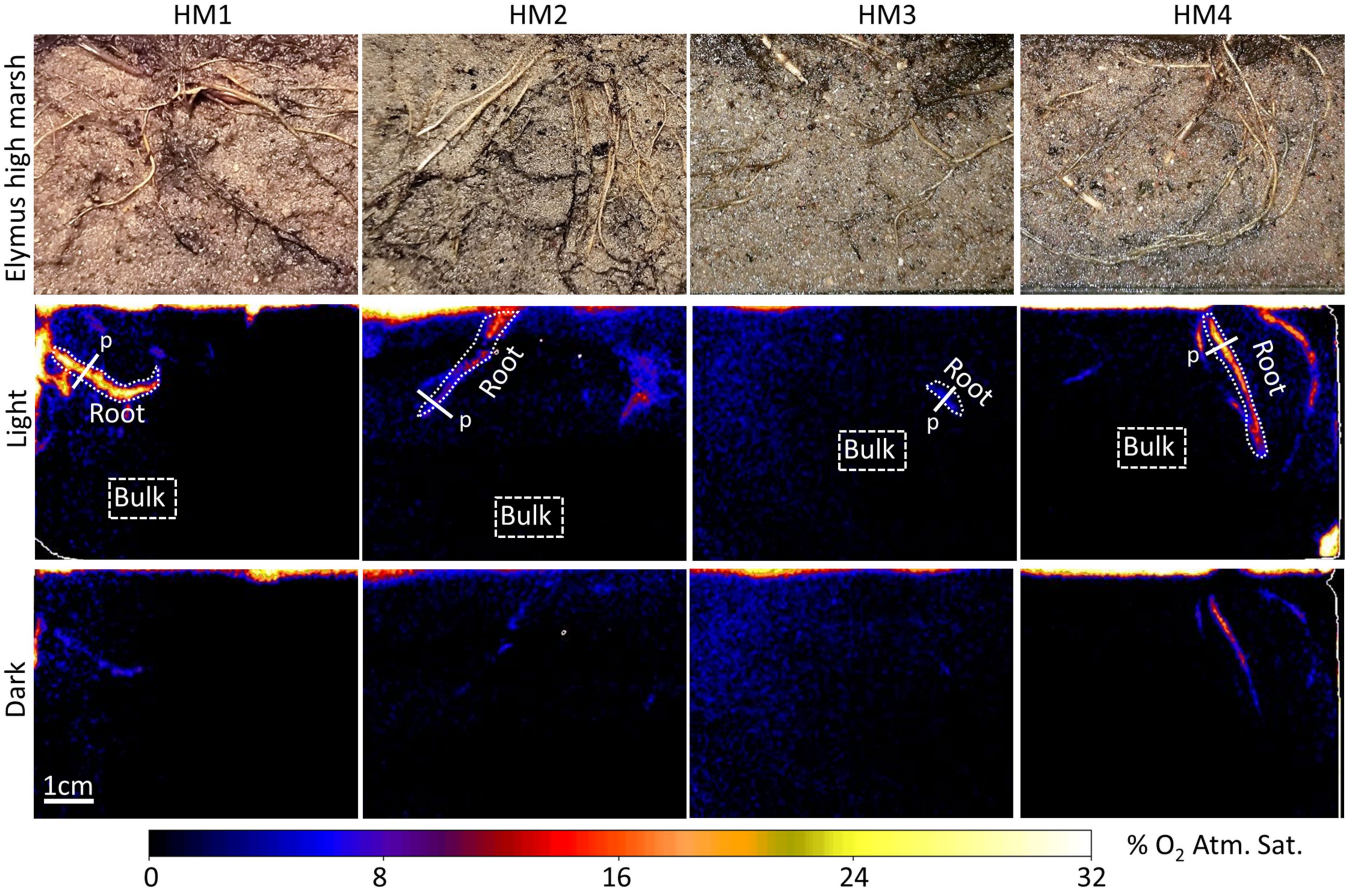
Planar optode images of the O_2_ distribution around selected roots of *E. athericus* – high-marsh ecotype from four independent samples (HM1–HM4), with and without illumination of the aboveground biomass. “Root” indicates specific oxic root zones, and “Bulk” indicates specific areas with bulk anoxic sediment selected for quantitative O_2_ measurements shown in Figure 4. “p” indicates the locations of the concentration profile shown in Figure 5.

Oxic root zones facilitated by plant-mediated O_2_ transport were found in both the low-marsh and the high-marsh ecotype of *Elymus* (Figures 2, 3). Although many roots were placed alongside of the rhizobox, only some of these roots were leaking O_2_ to the sediment. The size of the oxic root zones ranged from less than a millimeter on either side of a root to larger, centimeter-scale oxic roots zones, where multiple roots contributed to the oxygenation of the sediment.

O_2_ leakage was most pronounced in the low-marsh ecotype, where oxic root zones were generated around multiple roots in all four replicates (Figure 2). High variation was shown among samples; sediment oxygenation was particularly pronounced in LM1 and LM2, whereas in LM3 and LM4, plant-mediated sediment oxygenation was primarily found near the sediment surface with a few O_2_ leaking roots stretching into the sediment. Also, in the high-marsh ecotype (Figure 3), oxic root zones were found in all four replicates. Here, plant-mediated sediment oxygenation was primarily associated with roots near the sediment surface. In replicate HM1, HM2, and HM4, oxic root zones were found stretching from the sediment surface into the sediment, whereas HM3 showed very limited sediment oxygenation. Despite the reduction in the size and O_2_ concentration during dark periods, the oxic root zones remained visibly distinguishable from the bulk anoxic sediment in the optode images, although they were relatively faint due to low O_2_ concentrations (Figure 2 – dark and Figure 3 – dark). This demonstrated that *Elymus* is able to maintain oxic conditions around its roots, even without photosynthetic O_2_ production.

### Impact of Light Availability on the Temporal Variation of Oxic Root Zones

**Figure 4:**
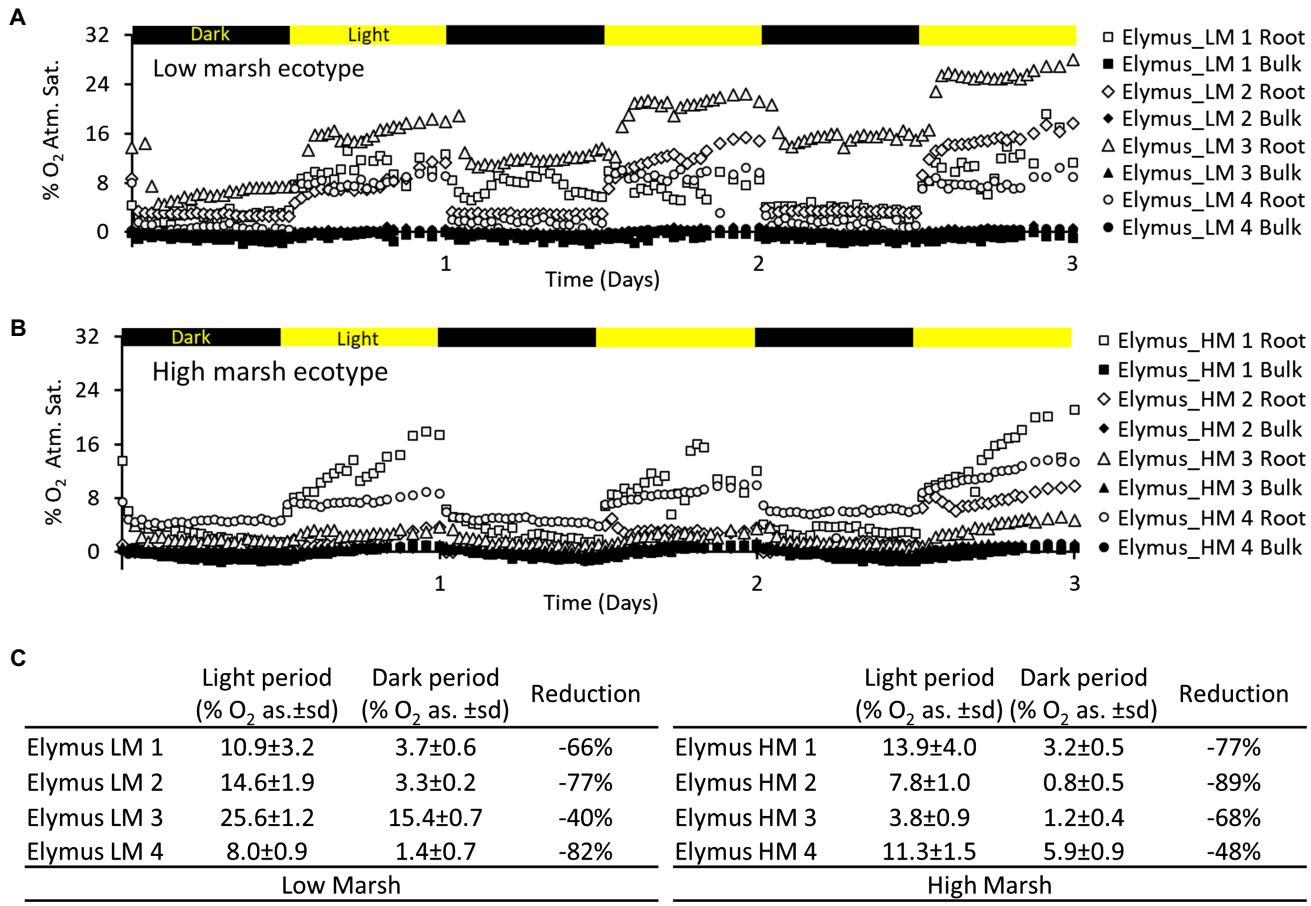
Oxygen dynamics in selected oxic root zones of the *E. athericus* – low-marsh ecotype **(A)** and high-marsh ecotype **(B)** during three light–dark periods (12 h/12 h). The location of the oxic root zones is depicted in the optode images of Figures 2, 3. **(C)** Table showing O_2_ concentrations [% O_2_ atm. sat.(as.)] in selected root zones of the low- and high-marsh ecotype, calculated as an average of the light period (2.5–3 d) and the preceding dark period (2–2.5 d), respectively; ±sd refers to the standard deviation (*n* = 24). “Reduction” shows the percentage reduction in the average concentration of O_2_ between light and dark periods.

The light conditions of the aboveground biomass markedly affected the belowground oxygenation of the rhizosphere. This was observed in the low-marsh and high-marsh ecotype (Figure 4). The average O_2_ concentration of the oxic root zones immediately decreased when light exposure of the aboveground biomass was turned off and immediately increased again when light exposure was turned back on. The reduction in the average O_2_ concentration during darkness was highly variable, ranging from 40 to 82% reduction in the low-marsh ecotype and 48–89% in the high-marsh ecotype. Despite the reduction in O_2_ concentration during dark periods, the oxic concentrations remained higher in the oxic root zone than in the bulk anoxic sediment (Figure 4). There was no statistical difference (*t*-test) between the low-marsh and the high-marsh ecotype in regard to O_2_ concentration during the light (*t* = 1.26, *p* = 0.26) and the dark period (*t* = 0.93, *p* = 0.39) or the reduction in O_2_ between light and dark periods (*t* = 0.33, *p* = 0.75) (*t*-tests based on data in Figure 4C).

### Impact of Light Availability on the Spatial Variation of Oxic Root Zones

**Figure 5:**
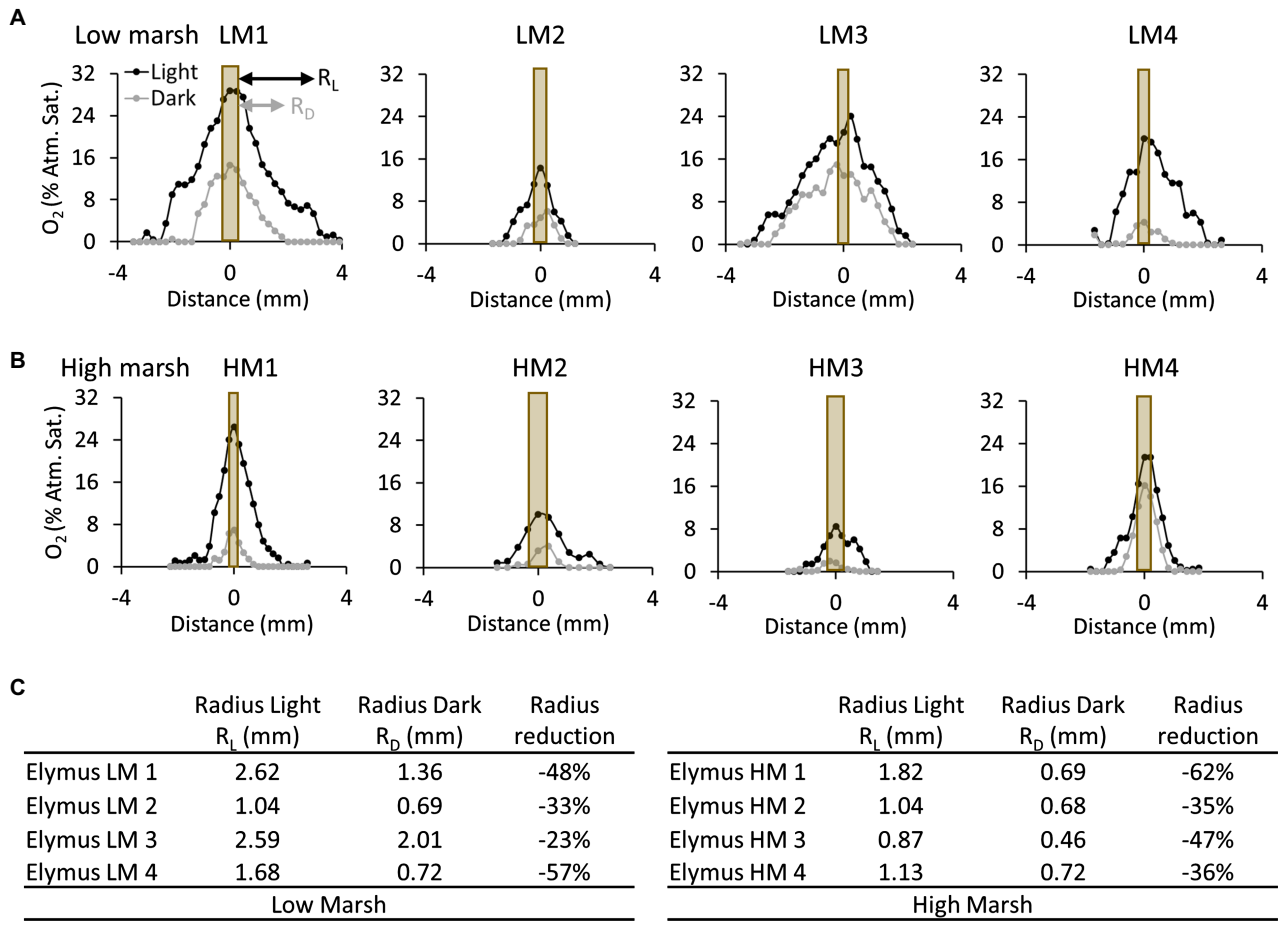
Oxygen concentration profiles measured perpendicular to selected roots of the *E. athericus* low-marsh ecotype **(A)** and high-marsh ecotype **(B)**. The locations of the profiles are depicted in Figures 2, 3. The orange bar shows the position of the root relative to the O_2_ profile, and the width of the bar represents the relative width of the root investigated. The profiles were measured during the last light cycle (2.5–3 d) and dark cycle (2–2.5 d), respectively, at the time point, when the oxygen concentration at the root surface was highest. **(C)** Table showing the radius of oxic root zones measured from the root surface to the bulk anoxic sediment in the low- and high-marsh ecotype during light and dark periods, respectively.

The spatial distribution of the oxic root zones in the low-marsh ecotype and high-marsh ecotype was affected by changing light conditions, diminishing when the light exposure of the aboveground biomass was turned off (Figure 5). The radius of the oxic root zones measured from the O_2_ profiles was markedly reduced by 23–57% in the low-marsh ecotype and 35–62% in the high-marsh ecotype. Despite the reduction in the width of the oxic root zones, the sediment remained oxic in the immediate vicinity of the roots during dark periods, and the sediment at the root surface never became fully anoxic (Figure 5). There was no statistical difference (*t*-test) between the low-marsh and the high-marsh ecotype in regard to the radius of the oxic root zone during light (*t* = 1.76, *p* = 0.13) and dark (*t* = 1.75, *p* = 0.13) periods or in the maximum O_2_ concentration at the root surface (*t* = 0.48, *p* = 0.65) (*t*-tests based on data in Figure 5C).

## Discussion

Oxic root zones were found in the low-marsh and high-marsh ecotype of *Elymus*. This clearly demonstrated that both ecotypes are genetically disposed to develop roots with the capacity to transport O_2_ belowground and oxygenate the sediment. In the optode images, the low-marsh ecotype displayed more and larger oxic root zones and with higher O_2_ concentrations below the sediment surface (Figures 2, 3). However, the radius of individual oxic root zones, measured around single roots, showed no difference between ecotypes and ranged from 0.9 to 2.6 mm under illuminated conditions. Hence, our study could not determine any clear distinction in the sediment oxygenation capacity between the two ecotypes. The here observed size range is similar to oxic root zones found in other wetland plants, such as *Spartina anglica* (Koop-Jakobsen et al., 2017) and rice (*Oryza sativa*; Larsen et al., 2015), and in aquatic macrophytes, such as *Ruppia maritima* (Jovanovic et al., 2015) and *Vallisneria spiralis* (Han et al., 2016). In cases where O_2_ leaking roots were overlapping (Figure 2; LM2 and LM3), larger oxic roots zones developed due to the cumulative contribution, causing O_2_ to build up in the sediment. This is often shown in aquatic plants where O_2_ leakage occurs from larger parts of the roots, such as the freshwater isoetid *Lobelia dortmanna* (Lenzewski et al., 2018) and rice plants (*O. sativa*; Larsen et al., 2015).

### Spatial Variation in Oxygen Leakage from *Elymus* Roots

In many wetland plants, suberin and lignin deposits in the hypodermis/exodermis prevent O_2_ leakage from the root into the sediment (Ejiri and Shiono, 2019) as well as intrusion of reduced phytotoxins from the sediment. Plants that develop this O_2_-loss barrier are often capable of long-distance O_2_ transportation to root tissues deep below the sediment surface, and due to the barrier formation, sediment oxygenation is usually restricted to small areas near the root tips (Colmer, 2003). In the O_2_-leaking roots observed in *Elymus*, there was no clear spatial restriction of O_2_ loss across the rhizodermis, and O_2_ leakage occurred over larger parts of the roots. Therefore, *Elymus* O_2_ transport and sediment oxygenation are likely to be limited to the topsoil. Mozdzer et al. (2016) argue that the capacity to develop a deep-reaching root system represents a key trait in many invasive wetland plants that enables them to utilize nutrients below the rooting zone of native plants. However, our results suggest that this invasion strategy may not play a critical role in the spread of *Elymus*.

Sediment phytotoxins, such as low molecular weight carboxylic acids, sulfides, or reduced iron, are known to trigger the formation of a barrier against O_2_ loss (Pedersen et al., 2021). As *in situ* phytotoxin concentrations may be higher than those generated in the soils used in our experimental setup, additional research on barrier formation is needed to confirm this notion under field conditions in the waterlogged low marsh.

It is noteworthy that a majority of the roots visible in the rhizobox images (Figures 2, 3), which were monitored with the planar optode system, did not leak O_2_ at all. Roots without O_2_ leakage were present both in the low-marsh and high-marsh ecotype. This demonstrates that the sediment-oxygenation capability varied among roots, which can be caused either by insufficient connectivity between the individual roots and the aboveground O_2_ resources or by reduction of the permeability of roots, for example, due to the formation of an O_2_-loss barrier in the hypodermis/exodermis or the accumulation of metal plaques on the root surface (Povidisa et al., 2009). The latter is particularly shown in older roots. In this study, roots at various life stages were present in the optode images. O_2_ leakage was most often associated with newer roots with a clear white root surface and roots with a light brown surface, possibly due to initial precipitation of metal oxides. Older roots colored dark-brown or black showed no or limited O_2_ leakage, possibly due to substantial metal-plaque formation given the observed color changes.

### Effect of Light on the Development of Oxic Root Zones

The oxic roots zones were highly affected by changing light conditions, resulting in a rapid decrease in the O_2_ level (Figure 4) and size (Figure 5), which occurred immediately (i.e., within 30 min) after the illumination of the aboveground biomass was turned off. However, despite a marked reduction of the oxic root zones, they remained distinguishable in the optode images obtained during dark periods (Figures 2, 3). The presence of oxic root zones during dark periods was also confirmed by the quantitative measures, where the O_2_ concentration in oxic root zones during dark periods was higher than the anoxic bulk sediment (Figure 4). Furthermore, the O_2_ profiles also showed the presence of O_2_ in darkness (Figure 5). This suggests that processes in the aboveground biomass triggered by light availability control belowground rhizosphere oxygenation. In light, stomata will open and provide full access to atmospheric O_2_, which will increase the flow of O_2_ to roots and rhizomes. In the nighttime, stomata are likely only to be partially opened and restrict access to atmospheric O_2_, which can account for the observed decrease in the O_2_ level of the oxic root zones during dark periods. However, the stomatal conductance was still sufficient to maintain the oxic root zones and prevent complete anoxia during darkness. Furthermore, photosynthetic O_2_ production of the aboveground biomass may increase the internal O_2_ concentration and contribute to the belowground O_2_ transport during the day. The light-inflicted fluctuations in the O_2_ concentration of the oxic root zones varied markedly among the individual roots, ranging from a 40 to 89% reduction between light and darkness. This demonstrates a high variability in the O_2_ leaking capacity among individual roots. In this study, where the sediment was homogenized removing any variation in the sediment O_2_ demand prior to the optode measurements, the variation observed in oxic root zones reflects variation in root properties rather than sediment properties. A variation in root properties can originate from (1) variation in the O_2_ transport capability from the atmosphere to roots, (2) continuous root growth which can alter the O_2_-leaking capacity during the experiment, and (3) from investigating roots at different levels of maturity expressing differences in barrier formation. Variation in oxic root zones and their development over time has previously been observed in similar optode studies with other aquatic plants (Koop-Jakobsen and Wenzhöfer, 2015; Koop-Jakobsen et al., 2018; Lenzewski et al., 2018).

### Methodology

The 2D image series provides detailed information on the spatial and temporal variation in the O_2_ content around individual roots over time, which was clearly demonstrated in this study (Figures 2, 3). In this way, the planar optode technology is an excellent tool for getting insight into root functions and root-soil interactions, while the roots are still embedded in the soil. However, it should be considered that planar optodes are an experimental tool. In this study, a sandy sediment was deliberately chosen, so the roots could easily be placed in direct contact with the optode foil. Freshwater, rather than saltwater, was used in this study to secure the growth of the individual plants under waterlogged conditions prior to the optode measurement. Growing in the high marsh, *Elymus* is adapted to low-range salinities (<10 ppt) but able to withstand both high salinities during floods and low salinity during rainfalls (Mueller et al., 2021). In our study, *Elymus* thrived in freshwater, and there was no evidence of hyposaline stress from using a low-salt environment.

Furthermore, the O_2_ leaking from roots is measured against an optode foil, which acts as a barrier, causing the halo of O_2_ around the roots to be slightly skewed compared to normal sediment conditions. Hence, the experimental conditions in this study differ from the natural sediment conditions in the field. Nevertheless, our optode experiments clearly demonstrate that both the low-marsh and the high-marsh ecotype of *E. athericus* possess the specific trait that enables it to transport O_2_ from aboveground sources to its roots and oxygenate the rhizosphere during short-term waterlogging. However, elucidating the impact on the rhizosphere under natural conditions will require further studies *in situ*.

### Conclusion and Perspective

Oxic root zones were found in all investigated plants of the low-marsh ecotype. Consequently, we accept our first hypothesis that the low-marsh ecotype possesses the ability to transport O_2_ from aboveground sources to its roots and further into the sediment. Oxic root zones were also clearly detectable in all of the investigated plants of the high-marsh ecotype. Hence, we reject our second hypothesis stating that the capability of transporting O_2_ is limited in the high-marsh ecotype. Plant-mediated sediment oxygenation is a key trait for aquatic plants facing waterlogged sediment conditions, and this trait is a prerequisite for *Elymus* to spread into the low marsh, potentially making it competitive with other species well adapted for living in waterlogged sediments, such as *Spartina*.

In a world with accelerating sea-level rise, salt marshes will experience more frequent flooding and longer times with waterlogged sediment conditions. As shown in this study, *Elymus* has the ability to improve the chemical environment of its rhizosphere through plant-mediated sediment oxygenation, and thereby, it can be a highly competitive species in a future, wetter salt-marsh environment. This can significantly impact the ecosystem services that the salt marshes provide in terms of coastal protection, carbon storage, and nutrient retention, and further studies are needed on the impact of alterations of the plant community composition caused by external factors, such as climate change.

## Data Availability Statement

The raw data supporting the conclusions of this article will be made available by the authors, without undue reservation.

## Author Contributions

KK-J and PM conceived the research idea. KK-J designed and performed the experiment, analyzed the data, and wrote the manuscript. RM provided the equipment and developed new software applications for image analysis. RM and PM edited the manuscript. All authors contributed to the article and approved the submitted version.

### Conflict of Interest

The sensor company, PreSens Precision Sensing GmbH, Regensburg, Germany, provided the planar optode equipment for this study. PreSens GmbH had no restrictive rights in regard to this publication beyond those entitled by their co-authorship. RJM was employed by company PreSens Precision Sensing GmbH.

The remaining authors declare that the research was conducted in the absence of any commercial or financial relationships that could be construed as a potential conflict of interest.
